# Instantaneous Non-Linear Processing by Pulse-Coupled Threshold Units

**DOI:** 10.1371/journal.pcbi.1000929

**Published:** 2010-09-09

**Authors:** Moritz Helias, Moritz Deger, Stefan Rotter, Markus Diesmann

**Affiliations:** 1 RIKEN Brain Science Institute, Wako City, Japan; 2 Bernstein Center for Computational Neuroscience, Freiburg, Germany; 3 Computational Neuroscience Lab, Faculty of Biology, Albert-Ludwig University, Freiburg, Germany; 4 Brain and Neural Systems Team, RIKEN Computational Science Research Program, Wako City, Japan; University College London, United Kingdom

## Abstract

Contemporary theory of spiking neuronal networks is based on the linear response of the integrate-and-fire neuron model derived in the diffusion limit. We find that for non-zero synaptic weights, the response to transient inputs differs qualitatively from this approximation. The response is instantaneous rather than exhibiting low-pass characteristics, non-linearly dependent on the input amplitude, asymmetric for excitation and inhibition, and is promoted by a characteristic level of synaptic background noise. We show that at threshold the probability density of the potential drops to zero within the range of one synaptic weight and explain how this shapes the response. The novel mechanism is exhibited on the network level and is a generic property of pulse-coupled networks of threshold units.

## Author Summary

Our work demonstrates a fast-firing response of nerve cells that remained unconsidered in network analysis, because it is inaccessible by the otherwise successful linear response theory. For the sake of analytic tractability, this theory assumes infinitesimally weak synaptic coupling. However, realistic synaptic impulses cause a measurable deflection of the membrane potential. Here we quantify the effect of this pulse-coupling on the firing rate and the membrane-potential distribution. We demonstrate how the postsynaptic potentials give rise to a fast, non-linear rate transient present for excitatory, but not for inhibitory, inputs. It is particularly pronounced in the presence of a characteristic level of synaptic background noise. We show that feed-forward inhibition enhances the fast response on the network level. This enables a mode of information processing based on short-lived activity transients. Moreover, the non-linear neural response appears on a time scale that critically interacts with spike-timing dependent synaptic plasticity rules. Our results are derived for biologically realistic synaptic amplitudes, but also extend earlier work based on Gaussian white noise. The novel theoretical framework is generically applicable to any threshold unit governed by a stochastic differential equation driven by finite jumps. Therefore, our results are relevant for a wide range of biological, physical, and technical systems.

## Introduction

Understanding the dynamics of single neurons, recurrent networks of neurons, and spike-timing dependent synaptic plasticity requires the quantification of how a single neuron transfers synaptic input into outgoing spiking activity. If the incoming activity has a slowly varying or constant rate, the membrane potential distribution of the neuron is quasi stationary and its steady state properties characterize how the input is mapped to the output rate. For fast transients in the input, time-dependent neural dynamics gains importance. The integrate-and-fire neuron model [1] can efficiently be simulated [2], [3] and well approximates the properties of mammalian neurons [4]–[6] and more detailed models [7]. It captures the gross features of neural dynamics: The membrane potential is driven by synaptic impulses, each of which causes a small deflection that in the absence of further input relaxes back to a resting level. If the potential reaches a threshold, the neuron emits an action potential and the membrane potential is reset, mimicking the after-hyperpolarization.

The analytical treatment of the threshold process is hampered by the pulsed nature of the input. A frequently applied approximation treats synaptic inputs in the diffusion limit, in which postsynaptic potentials are vanishingly small while their rate of arrival is high. In this limit, the summed input can be replaced by a Gaussian white noise current, which enables the application of Fokker-Planck theory [8], [9]. For this approximation the stationary membrane potential distribution and the firing rate are known exactly [8], [10], [11]. The important effect of synaptic filtering has been studied in this limit as well; modelling synaptic currents as low-pass filtered Gaussian white noise with non-vanishing temporal correlations [12]–[15]. Again, these results are strictly valid only if the synaptic amplitudes tend to zero and their rate of arrival goes to infinity. For finite incoming synaptic events which are excitatory only, the steady state solution can still be obtained analytically [16], [17] and also the transient solution can efficiently be obtained by numerical solution of a population equation [18]. A different approach takes into account non-zero synaptic amplitudes to first calculate the free membrane potential distribution and then obtain the firing rate by solving the first passage time problem numerically [19]. This approach may be extendable to conductance based synapses [20]. Exact results for the steady state have so far only been presented for the case of exponentially distributed synaptic amplitudes [21].

**Figure 1:**
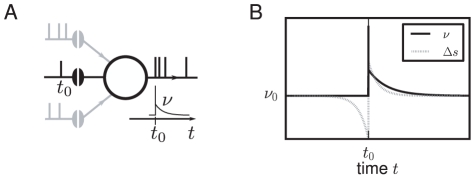
Firing rate response 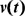 to a synaptic input. A The neuron receives excitatory and inhibitory background events (gray spikes) from many synapses. We focus on one such incoming synapse that carries a synaptic impulse at 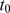 (black spike). B The firing rate of the neuron triggered on this event shows a deflection (black solid curve) from the base rate 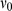. If the synaptic efficacy obeys a spike-timing dependent learning rule, the synaptic weight changes by 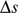 according to the relative timing of the presynaptic spike and the action potentials emitted by the neuron (gray dotted curve indicates typical dependence). The time-averaged change in synaptic weight depends on the integrated pointwise product of both curves. Their relative position depends on the axonal and dendritic delays involved (neglected for simplicity in this schematic).

The spike threshold renders the model an extremely non-linear unit. However, if the synaptic input signal under consideration is small compared to the total synaptic barrage, a linear approximation captures the main characteristics of the evoked response. In this scenario all remaining inputs to the neuron are treated as background noise (see Figure 1A). Calculations of the linear response kernel in the diffusion limit suggested that the integrate-and-fire model acts as a low-pass filter [22]. Here spectrum and amplitude of the synaptic background input are decisive for the transient properties of the integrate-and-fire model: in contrast to white noise, low-pass filtered synaptic noise leads to a fast response in the conserved linear term [12]. Linear response theory predicts an optimal level of noise that promotes the response [23]. In the framework of spike-response models, an immediate response depending on the temporal derivative of the postsynaptic potential has been demonstrated in the regime of low background noise [24]. The maximization of the input-output correlation at a finite amplitude of additional noise is called stochastic resonance and has been found experimentally in mechanoreceptors of crayfish [25], in the cercal sensory system of crickets [26], and in human muscle spindles [27]. The relevance and diversity of stochastic resonance in neurobiology was recently highlighted in a review article [28].

Linear response theory enables the characterization of the recurrent dynamics in random networks by a phase diagram [22], [29]. It also yields approximations for the transmission of correlated activity by pairs of neurons in feed-forward networks [30], [31]. Furthermore, spike-timing dependent synaptic plasticity is sensitive to correlations between the incoming synaptic spike train and the firing of the neuron (see Figure 1), captured up to first order by the linear response kernel [32]–[38]. For neuron models with non-linear membrane potential dynamics, the linear response properties [39], [40] and the time-dependent dynamics can be obtained numerically [41]. Afferent synchronized activity, as it occurs e.g. in primary sensory cortex [42], easily drives a neuron beyond the range of validity of the linear response. In order to understand transmission of correlated activity, the response of a neuron to fast transients with a multiple of a single synaptic amplitude [43] hence needs to be quantified.

In simulations of neuron models with realistic amplitudes for the postsynaptic potentials, we observed a systematic deviation of the output spike rate and the membrane potential distribution from the predictions by the Fokker-Planck theory modeling synaptic currents by Gaussian white noise. We excluded any artifacts of the numerics by employing a dedicated high accuracy integration algorithm [44], [45]. The novel theory developed here explains these observations and lead us to the discovery of a new early component in the response of the neuron model which linear response theory fails to predict. In order to quantify our observations, we extend the existing Fokker-Planck theory [46] and hereby obtain the mean time at which the membrane potential first reaches the threshold; the mean first-passage time. The advantage of the Fokker-Planck approach over alternative techniques has been demonstrated [47]. For non-Gaussian noise, however, the treatment of appropriate boundary conditions for the membrane potential distribution is of utmost importance [48]. In the results section we develop the Fokker-Planck formalism to treat an absorbing boundary (the spiking threshold) in the presence of non-zero jumps (postsynaptic potentials). For the special case of simulated systems propagated in time steps, an analog theory has recently been published by the same authors [49], which allows to assess artifacts introduced by time-discretization.

Our theory applied to the integrate-and-fire model with small but finite synaptic amplitudes [1], introduced in section “The leaky integrate-and-fire model”, quantitatively explains the deviations of the classical theory for Gaussian white noise input. After reviewing the diffusion approximation of a general first order stochastic differential equation we derive a novel boundary condition in section “Diffusion with finite increments and absorbing boundary”. We then demonstrate in section “Application to the leaky integrate-and-fire neuron” how the steady state properties of the model are influenced: the density just below threshold is increased and the firing rate is reduced, correcting the preexisting mean first-passage time solution [10] for the case of finite jumps. Turning to the dynamic properties, in section “Response to fast transients” we investigate the consequences for transient responses of the firing rate to a synaptic impulse. We find an instantaneous, non-linear response that is not captured by linear perturbation theory in the diffusion limit and that displays marked stochastic resonance. On the network level, we demonstrate in section “Dominance of the non-linear component on the network level” that the non-linear fast response becomes the most important component in case of feed-forward inhibition. In the discussion we consider the limitations of our approach, mention possible extensions and speculate about implications for neural processing and learning.

## Model

### The leaky integrate-and-fire model

Consider a leaky integrate-and-fire model [1] with membrane time constant 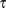 and resistance 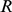 receiving excitatory and inhibitory synaptic inputs, as they occur in balanced neural networks [50]. We aim to obtain the mean firing rate 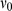 and the steady state membrane potential distribution 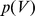. The input current 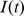 is modeled by point events 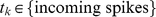, drawn from homogeneous Poisson processes with rates 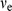 and 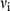, respectively. The membrane potential is governed by the differential equation 
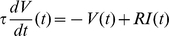
. An excitatory spike causes a jump of the membrane potential by 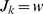, an inhibitory spike by 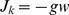, so 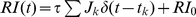, where 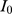 is a constant background current. Whenever 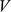 reaches the threshold 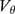, the neuron emits a spike and the membrane potential is reset to 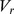, where it remains clamped for the absolute refractory time 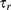. The approach we take is to modify the existing Fokker-Planck theory in order to capture the major effects of the finite jumps. To this end, we derive a novel boundary condition at the firing threshold for the steady state membrane potential distribution of the neuron. We then solve the Fokker-Planck equation obtained from the standard diffusion approximation [8], [10], [11], [22], [23] given this new condition.

## Results

### Diffusion with finite increments and absorbing boundary

The membrane potential of the model neuron follows a first order stochastic differential equation. Therefore, in this section we consider a general first order stochastic differential equation driven by point events. In order to distinguish the dimensionless quantities in this section from their counterparts in the leaky integrate-and-fire model, we denote the rates of the two incoming Poisson processes by 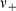 (excitation) and 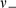 (inhibition). Each incoming event causes a finite jump 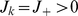 (the excitatory synaptic weight) for an increasing event and 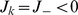 (the inhibitory synaptic weight) for a decreasing event. The stochastic differential equation takes the form
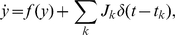
(1)where 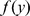 captures the deterministic time evolution of the system (with 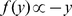 for the leaky integrate-and-fire neuron). We follow the notation in [46] and employ the Kramers-Moyal expansion with the infinitesimal moments 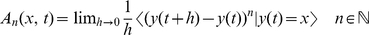. The first and second infinitesimal moment evaluate to 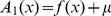 and 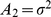, where we introduced the shorthand 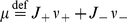 and 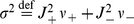. The time evolution of the probability density 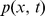 is then governed by the Kramers-Moyal expansion, which we truncate after the second term to obtain the Fokker-Planck equation
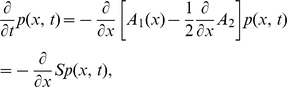
(2)where 
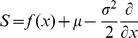
 denotes the probability flux operator.

In the presence of an absorbing boundary at 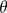, we need to determine the resulting boundary condition for the stationary solution of (2). Without loss of generality, we assume the absorbing boundary at 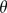 to be the right end of the domain. A stationary solution exists, if the probability flux exiting at the absorbing boundary is reinserted into the system. For the example of an integrate-and-fire neuron, reinsertion takes place due to resetting the neuron to the same potential after each threshold crossing. This implies a constant flux 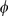 through the system between the point of insertion 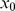 and threshold 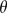. Rescaling the density by this flux as 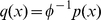 results in the stationary Focker-Planck equation, which is a linear inhomogeneous differential equation of first order
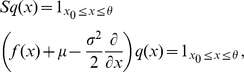
(3)with 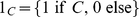.

First we consider the diffusion limit, in which the rate of incoming events diverges, while the amplitude of jumps goes to zero, such that mean 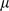 and fluctuations 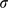 remain constant. In this limit, the Kramers-Moyal expansion truncated after the second term becomes exact [51]. This route has been taken before by several authors [8], [22], [23], here we review these results to consistently present our extension of the theory. In the above limit equation (3) needs to be solved with the boundary conditions
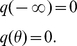
Moreover, a finite probability flux demands the density to be a continuous function, because of the derivative in the flux operator 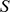. In particular, the solution must be continuous at the point of flux insertion 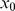 (however, the first derivative is non-continuous at 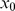 due to the step function in the right hand side of (3)). Continuity especially implies a vanishing density at threshold 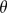. Once the solution of (3) is found, the normalization condition 
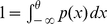
 determines the stationary flux 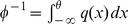.

Now we return to the problem of finite jumps. We proceed along the same lines as in the diffusion limit, seeking the stationary solution of the Fokker-Planck equation (2). We keep the boundary conditions at 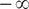 and at 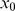 as well as the normalization condition as before, but we need to find a new self-consistent condition at threshold 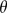, because the density does not necessarily have to vanish if the rate of incoming jumps is finite. The main assumption of our work is that the steady state solution satisfies the stationary Fokker-Planck equation (3) based on the diffusion approximation within the interval 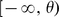, but not necessarily at the absorbing boundary 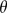, where the solution might be non-continuous. To obtain the boundary condition, we note that the flux over the threshold has two contributions, the deterministic drift and the positive stochastic jumps crossing the boundary
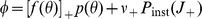
(4)

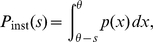
(5)with 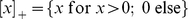. To evaluate the integral in (5), for small 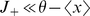 we expand 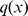 into a Taylor series around 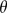. This is where our main assumption enters: we assume that the stationary Fokker-Planck equation (3) for 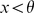 is a sufficiently accurate characterization of the jump diffusion process. We solve this equation for 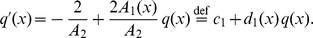 It is easy to see by induction, that the function and all its higher derivatives 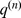, 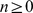 can be written in the form 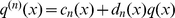, whose coefficients for 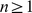 obey the recurrence relation
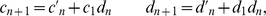
(6)with the additional values 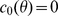 and 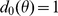, as 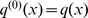 denotes the function itself. Inserting the Taylor series into (5) and performing the integration results in
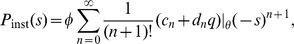
(7)which is the probability mass moved across threshold by a perturbation of size 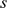 and hence also quantifies the instantaneous response of the system. After dividing (4) by 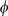 we solve for 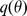 to obtain the Dirichlet boundary condition
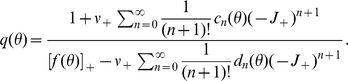
(8)


If 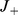 is small compared to the length scale on which the probability density function varies, the probability density near the threshold is well approximated by a Taylor polynomial of low degree; throughout this work, we truncate (7) and (12) at 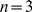. The boundary condition (8) is consistent with 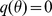 in the diffusion limit, in which the rate of incoming jumps diverges, while their amplitude goes to zero, such that the first (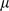) and second moment (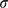) stay finite. This can be seen by scaling 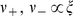, 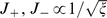, with 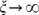 such that the mean 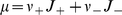 is kept constant [51]. Inserting this limit in (8), we find
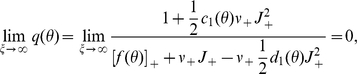
(9)since 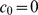, 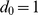 and 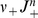 vanishes for 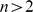, 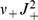 is bounded and 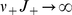.

The general solution of the stationary Fokker-Planck equation (3) is a sum of a homogeneous solution 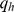 that satisfies 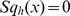 and a particular solution with 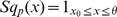. The homogeneous solution is 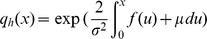, where we fixed the integration constant by chosing 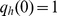. The particular solution can be obtained by variation of constants and we chose it to vanish at the threshold 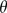 as 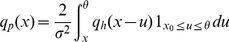. The complete solution is a linear combination, where the prefactor 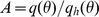 is determined by the boundary condition (8) in the case of finite jumps, or by 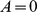 for Gaussian white noise
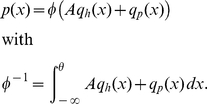



The normalization condition determines the as yet unknown constant probability flux 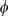 through the system.

### Application to the leaky integrate-and-fire neuron

**Figure 2:**
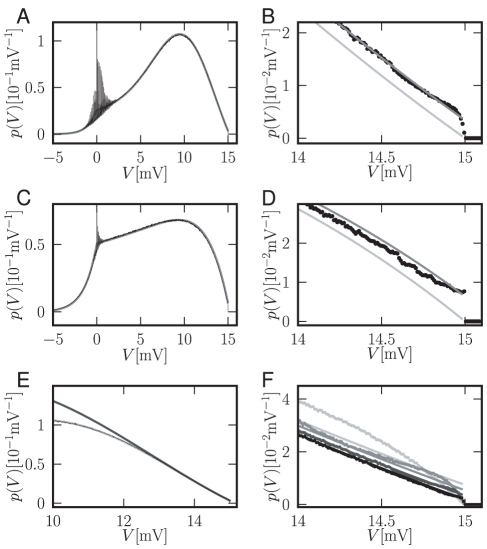
Finite synaptic potentials distort the stationary membrane potential density 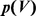. A Black thin curve: direct simulation. Parameters 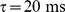, 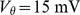, 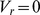, 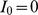, 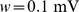, 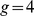, 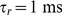. Incoming spike rates 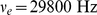, 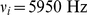 (corresponding to 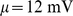 and 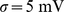). Histogram binned with 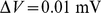, entries connected by straight lines. Gray: novel approximation 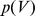 given by (14). B Magnification of A around spike threshold, simulated data displayed as dots. Light gray: solution in diffusion limit of [22]. C,D Density for supra-threshold current 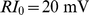 and incoming rates 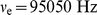, 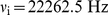 (corresponding to 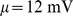 and 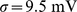). Other parameters and gray code as in A,B. E Approximation of the density by a cubic polynomial near threshold. Solid light gray curve: analytical result 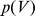 given by (14), superimposed black thin curve: direct simulation. Dark gray solid curve: cubic polynomial approximating the density around 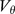 using the Taylor expansion (6). Parameters as in A. F Membrane potential distribution near threshold for synaptic amplitudes 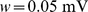 (black), 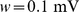 (dark gray), 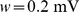 (gray), 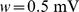 (light gray). Other parameters as in A.

**Figure 3:**
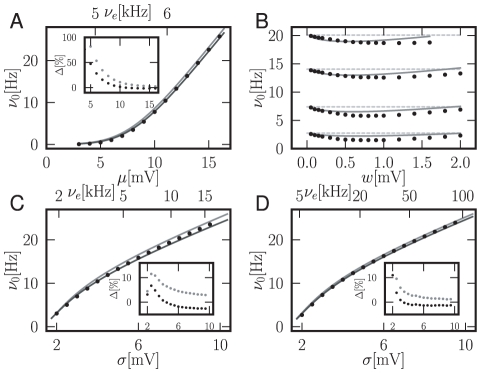
Correction of the firing rate. A Analytical firing rate compared to simulation in dependence of the mean drive 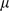. Black: Analytical solution (13), light gray: firing rate in diffusion limit [10], [29]. Inset shows relative error 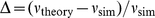 of theory compared to direct simulation as percentage, black dots: error of analytical expression (13), light gray dots: error of rate in diffusion limit. Parameters 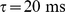, 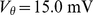, 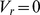, 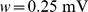, 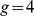, 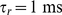. 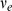 (given on top abcissa) and 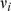 chosen to realize the mean input 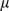 as given by the bottom abscissa and fluctuations 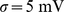. B Analytical firing rate compared to simulation depending on the size of synaptic jumps 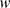 for fixed 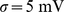 and 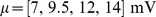 (from bottom to top). Light gray dashed line: firing rate in diffusion limit [10], [29], other gray code as in A. C Analytical firing rate compared to simulation as a function of the fluctations 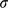 at fixed mean drive 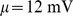. Same gray code and other parameters as in A. D Analytical firing rate compared to simulation depending on the fluctation 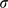 at fixed 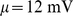 for smaller synaptic amplitude 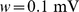. Same gray code and other parameters as in A.

We now apply the theory developed in the previous section to the leaky integrate-and-fire neuron with finite postsynaptic potentials. Due to synaptic impulses, the membrane potential drifts towards 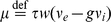 and fluctuates with the diffusion constant 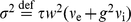. This suggests to choose the natural units 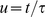 for the time and 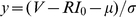 for the voltage to obtain the simple expressions 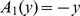 for the drift- and 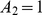 for the diffusion-term in the Fokker-Planck operator (2). The probability flux operator (2) is then given as 
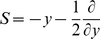
. In the same units the stationary probability density scaled by the flux reads 
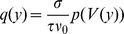
 where 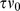 is the flux 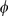 corresponding to the firing rate in units of 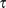. As 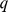 is already scaled by the flux, application of the flux operator 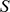 yields unity between reset 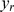 and threshold 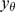 and zero outside
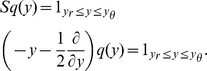
(10)The steady state solution of this stationary Fokker-Planck equation
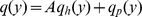
(11)is a linear superposition of the homogeneous solution 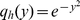 and the particular solution 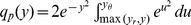. The latter is chosen to be continuous at 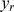 and to vanish at 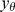. Using the recurrence (6) for the coeffcients of the Taylor expansion of the membrane potential density, we obtain 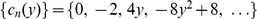 and 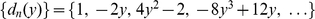, where 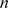 starts from 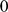. The first important result of this section is the boundary value 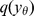 of the density at the threshold following from (8) as
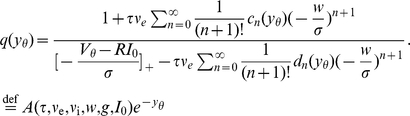
(12)The constant 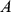 in (11) follows from 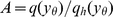. The second result is the steady state firing rate 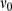 of the neuron. With 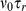 being the fraction of neurons which are currently refractory, we obtain the rate from the normalization condition of the density 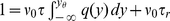 as
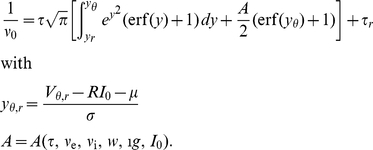
(13)The normalized steady state solution Figure 2A therefore has the complete form
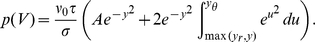
(14)
Figure 2B,D shows the steady state solution near the threshold obtained by direct simulation to agree much better with our analytical approximation than with the theory for Gaussian white noise input. Even for synaptic amplitudes (here 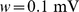) which are considerably smaller than the noise fluctuations (here 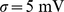), the effect is still well visible. The oscillatory deviations with periodicity 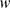 close to reset 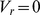 observable in Figure 2A are due to the higher occupation probability of voltages that are integer multiples of a synaptic jump away from reset. The modulation washes out due to coupling of adjacent voltages by the deterministic drift as one moves away from reset. The oscillations at lower frequencies apparent in Figure 2A are due to aliasing caused by the finite bin width of the histogram (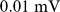). The synaptic weight is typically small compared to the length scale 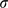 on which the probability density function varies. So the probability density near the threshold is well approximated by a Taylor polynomial of low degree; throughout this work, we truncate the series in (12) at 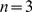. A comparison of this approximation to the full solution is shown in Figure 2E. For small synaptic amplitudes (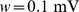 shown), below threshold and outside the reset region (Figure 2A,C) the approximation agrees with the simulation within its fluctuation. At the threshold (Figure 2B,D) our analytical solution assumes a finite value 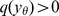 whereas the direct simulation only drops to zero on a very short voltage scale on the order of the synaptic amplitude. For larger synaptic weights (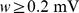, see Figure 2F), the density obtained from direct simulation exhibits a modulation on the corresponding scale. The reason is the rectifying nature of the absorbing boundary: A positive fluctuation easily leads to a threshold crossing and absorption of the state in contrast to negative fluctuations. Effectively, this results in a net drift to lower voltages within the width of the jump distribution caused by synaptic input, visible as the depletion of density directly below the threshold and an accumulation further away, as observed in Figure 2F. The second term (proportional to 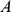) appearing in (13) is a correction to the well known firing rate equation of the integrate-and-fire model driven by Gaussian white noise [10]. Figure 3 compares the firing rate predicted by the new theory to direct simulation and to the classical theory. The classical theory consistently overestimates the firing rate, while our theory yields better accuracy. Our correction resulting from the new boundary condition becomes visible at moderate firing rates when the density slightly below threshold is sufficiently high. At low mean firing rates, the truncation of the Kramers-Moyal expansion employed in the Fokker-Planck description may contribute comparably to the error. Our approximation captures the dependence on the synaptic amplitude correctly for synaptic amplitudes of up to 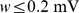 (Figure 3B). The insets in Figure 3C,D show the relative error of the firing rate as a function of the noise amplitude. As expected, the error increases with the ratio of the synaptic effect 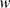 compared to the amplitude of the noise fluctuations 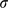. For low noise 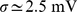, our theory reduces the relative error by a factor of 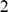 compared to the classical diffusion approximation.

### Response to fast transients

**Figure 4:**
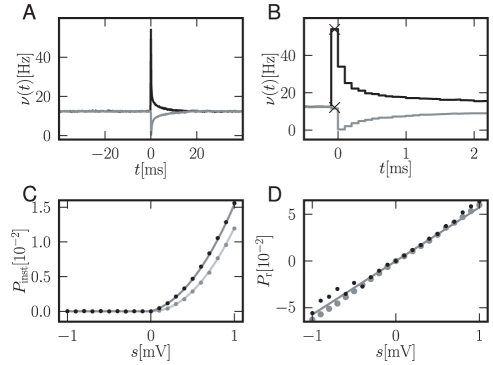
Firing rate response to a 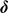-current perturbation. A Black: response to an additionally injected current 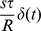 causing a voltage deflection of 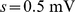 at 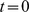, gray: 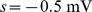. B Magnification of A. Due to binning of the histogram with bin size 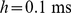, the immediate response contributes to the time bin 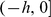 in the case of a positive perturbation only. Black crosses: analytical peak responses 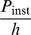 (7) for positive and negative perturbations. C Medium gray curve: instantaneous response 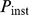 (7) as a function of 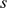 for finite weights 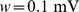. Black dots: direct simulation. Light gray curve: diffusion limit of (7). Medium gray dots: direct simulation of diffusion limit with temporal resolution 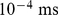. D Gray curve: integral response for finite weights (15). Black dots: direct simulation. Gray dots: direct simulation for Gaussian white noise background input. Simulated data averaged over 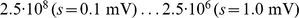 perturbation events. Other parameters as in Figure 2A.

**Figure 5:**
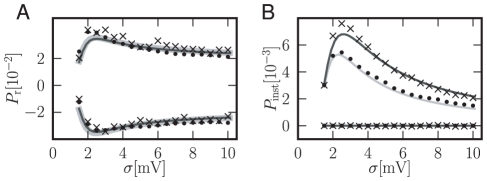
Noise dependence of response to a 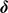-current perturbation. A Integrated response of the firing rate (15)of the integrate-and-fire model as a function of synaptic background noise 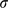. The upper traces are the responses to a positive perturbation of magnitude 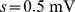, the lower to a perturbation of 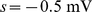. Black crosses: direct simulation with background noise of finite synaptic weights 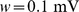. Black dots: direct simulation with Gaussian white background noise. Mid gray thin solid curve: analytical result using (16). Underlying thick light gray solid curve: analytical result for Gaussian white noise. B Instantaneous response depending on synaptic background noise 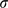. The upper trace is the response to a positive impulse of weight 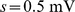, the lower one to a negative of 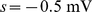. Gray code as in A. Mid gray solid curve: analytical peak response 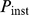 using (7) for noise of finite synaptic weights. Light gray solid curve: analytical peak response 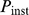 for Gaussian white background noise. Simulations averaged over 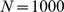 neurons for 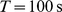. Incoming spike rates 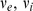 chosen to realize 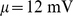 and 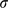 on the abscissa for synaptic weights 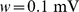 and 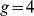.

We now proceed to obtain the response of the firing rate 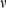 to an additional 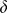-shaped input current 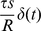. Such a current can be due to a single synaptic event or due to the synchronized arrival of several synaptic pulses. In the latter case, the effective amplitude of the summed inputs can easily exceed that of a single synapse. The fast current transient 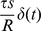 causes a jump 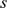 of the membrane potential at 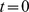 and (2) suggests to treat the incident as a time dependent perturbation of the mean input 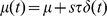. First, we are interested in the integral response 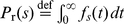 of the excess firing rate 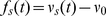. Since the perturbation has a flat spectrum, up to linear order in 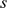 the spectrum of the excess rate is 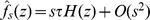, where 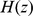 is the linear transfer function with respect to perturbing 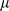 at Laplace frequency 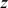. In particular, 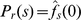. As 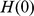 is the DC susceptibility of the system, we can express it up to linear order as 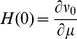. Hence,
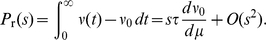
(15)We also take into account the dependence of 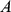 on 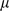 to calculate 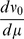 from (13) and obtain
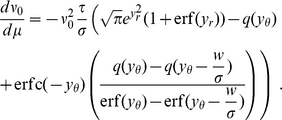
(16)
Figure 4D shows the integral response to be in good agreement with the linear approximation. This expression is consistent with the result in the diffusion limit 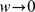: Here the last term becomes 
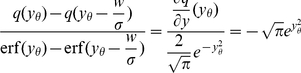
, where we used 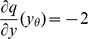, following from (10) with 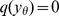. This results in 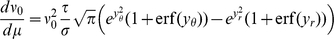, which can equivalently be obtained directly as the derivative of (13) with respect to 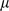 setting 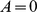. Taking the limit 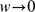, however, does not change significantly the integral response compared to the case of finite synaptic amplitudes (Figure 4D, Figure 5A).

The instantaneous response of the firing rate to an impulse-like perturbation can be quantified without further approximation. The perturbation shifts the probability density by 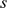 so that neurons with 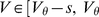] immediately fire. This results in the finite firing probability 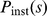 of the single neuron within infinitesimal time (5), which is zero for 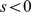. This instantaneous response has several interesting properties: For small 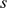 it can be approximated in terms of the value and the slope of the membrane potential distribution below the threshold (using (7) for 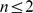), so it has a linear and a quadratic contribution in 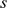. Figure 4A shows a typical response of the firing rate to a perturbation. The peak value for a positive perturbation agrees well with the analytical approximation (7) (Figure 4C). Even in the diffusion limit, replacing the background input by Gaussian white noise, the instantaneous response persists. Using the boundary condition 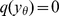 our theory is applicable to this case as well. Since the density just below threshold is reduced, (5) yields a smaller instantaneous response (Figure 4C, Figure 5B) which for positive 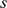 still exhibits a quadratic, but no linear, dependence.

The increasing and convex dependence of the response probability on the amplitude of the perturbation is a generic feature of neurons with subthreshold mean input that also persists in the case of finite synaptic rise time. In this regime, the membrane potential distribution has a mono-modal shape centered around the mean input, which is inherited from the underlying superposition of a large number of small synaptic impulses. The decay of the density towards the threshold is further enhanced by the probability flux over the threshold: a positive synaptic fluctuation easily leads to the emission of a spike and therefore to the absorption of the state at the threshold, depleting the density there. Consequently, the response probability 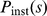 of the neuron is increasing and convex as long as the peak amplitude 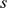 of the postsynaptic potential is smaller than the distance of the peak of the density to the threshold. It is increasing and concave beyond this point. At present the integrate-and-fire model is the simplest analytically tractable model with this feature.

The integral response (15) as well as the instantaneous response (5) both exhibit stochastic resonance; an optimal level of synaptic background noise 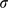 enhances the transient. Figure 5A shows this noise level to be at about 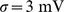 for the integral response. The responses to positive and negative perturbations are symmetric and the maximum is relatively broad. The instantaneous response in Figure 5B displays a pronounced peak at a similar value of 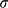. This non-linear response only exists for positive perturbations; the response is zero for negative ones. Though the amplitude is reduced in the case of Gaussian white noise background, the behavior is qualitatively the same as for noise with finite jumps. Stochastic resonance has been reported for the linear response to sinusoidal periodic stimulation [23]. Also for non-periodic signals that are slow compared to the neuron's dynamics an adiabatic approximation reveals stochastic resonance [52]. In contrast to the latter study, the rate transient observed in our work is the instantaneous response to a fast (Dirac 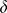) synaptic current.

### Dominance of the non-linear component at the network level

Due to the convex nature of the instantaneous response (Figure 4C) its relative contribution to the integral response increases with 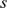. For realistic synaptic weights 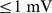 the contribution reaches 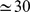 percent.

**Figure 6:**
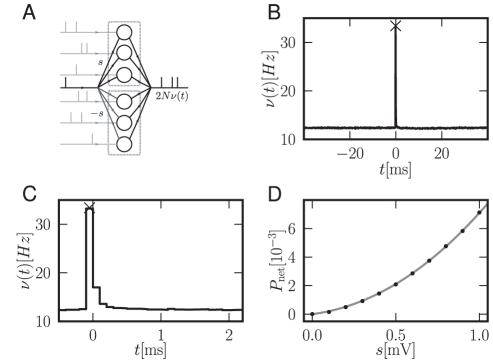
Dominance of non-linearity at the network level. A Two identical populations of 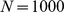 neurons each, receive uncorrelated background input (light gray spikes). At 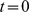 the neurons simultaneously receive an additional input of size 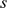 in the upper and 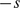 in the lower population (symbolized by black single spike). B Pooled response of the populations normalized by the number of neurons. Black cross: analytical instantaneous response 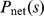. C Magnification of B. D 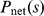 (black dots: direct simulation, gray curve: analytical result) as a function of 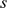. Other parameters as in Figure 4A.

An example network in which the linear non-instantaneous response cancels completely and the instantaneous response becomes dominant is shown in Figure 6A. At 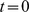 two populations of neurons simultaneously receive a perturbation of size 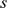 and 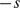 respectively. This activity may, for example, originate from a third pool of synchronous excitatory and inhibitory neurons. It may thus be interpreted as feed-forward inhibition. The linear contributions to the pooled firing rate response of the former two populations hence is zero. The instantaneous response, however, causes a very brief overshoot at 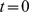 (Figure 6B). Figure 6C reveals that the response returns to baseline within 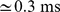. Figure 6D shows that the dependence of peak height 
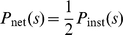
 on 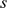 still exhibits the supra-linearity. The quite exact cancellation of the response for 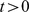 originates from the symmetry of the response functions for positive and negative perturbations in this interval (shown in Figure 4A,B). The pooled firing rate of the network is the sum of the full responses: the instantaneous response at 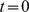 does not share the symmetry and hence does not cancel. This demonstrates that the result of linear perturbation theory is a good approximation for 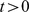 and that the instantaneous response at the single time point 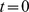 completes the characterization of the neuronal response.

## Discussion

In this work we investigate the effect of small, but non-zero synaptic impulses on the steady state and response properties of the integrate-and-fire neuron model. We obtain a more accurate description of the firing rate and the membrane potential distribution in the steady state than provided by the classical approximation of Gaussian white noise input currents [10]. Technically this is achieved by a novel hybrid approach combining a diffusive description of the membrane potential dynamics far away from the spiking threshold with an explicit treatment of threshold crossings by synaptic transients. This allows us to obtain a boundary condition for the membrane potential density at threshold that captures the observed elevation of density. Our work demonstrates that in addition to synaptic filtering, the granularity of the noise due to finite non-zero amplitudes does affect the steady state and the transient response properties of the neuron. Here, we study the effect of granularity using the example of a simple neuron model with only one dynamic variable. The quantitatively similar increase of the density close to threshold observed if low-pass filtered Gaussian white noise is used as a model for the synaptic current has a different origin. It is due to the absence of a diffusion term in the dynamics of the membrane potential [12], [13], [15]. The analytical treatment of finite synaptic amplitudes further allows us to characterize the probability of spike emission in response to synaptic inputs for neuron models with a single dynamical variable and renewal. Alternatively, this response can be obtained numerically from population descriptions [18], [39]–[41] or, for models with one or more dynamic variables and gradually changing inputs, in the framework of the refractory density approximation [15]. Here, we find that the response can be decomposed into a fast, non-linear and a slow linear contribution, as observed experimentally about a quarter of a century ago [53] in motor neurons of cat cortex in the presence of background noise. The existence of a fast contribution proportional to the temporal change of the membrane potential was predicted theoretically [54]. In the framework of the refractory density approach [15], the effective hazard function of an integrate-and-fire neuron also exhibits contributions to spike emission due to two distinct causes: the diffusive flow through the threshold and the movement of density towards the threshold. The latter contribution is proportional to the temporal change of the membrane potential and is corresponding to the instantaneous response reported here, but for the case of a gradually increasing membrane potential. Contemporary theory of recurrent networks so far has neglected the transient non-linear component of the neural response, an experimentally observed feature [53] that is generic to threshold units in the presence of noise. The infinitely fast rise of the postsynaptic potential in the integrate-and-fire model leads to the immediate emission of a spike with finite probability. For excitatory inputs, this probability depends supra-linearly on the amplitude of the synaptic impulse and it is zero for inhibitory impulses. The supra-linear increase for small positive impulse amplitudes relates to the fact that the membrane potential density decreases towards threshold: the probability to instantaneously emit a spike equals the integral of the density shifted over the threshold. The detailed shape of the density below threshold therefore determines the response properties. For Gaussian white noise synaptic background, the model still displays an instantaneous response. However, since in this case the density vanishes at threshold, the response probability to lowest order grows quadratically in the amplitude of a synaptic impulse. This is the reason why previous work based on linear response theory did not report on the existence of an instantaneous component when modulating the mean input and on the contrary characterized the nerve cell as a low-pass in this case [22], [23]. Modulation of the noise amplitude, however, has been shown to cause an instantaneous response in linear approximation in the diffusion limit [23], confirmed experimentally in real neurons [55]. While linear response theory has proven extremely useful to understand recurrent neural networks [29], the categorization of the integrate-and-fire neuron's response kernel as a low-pass is misleading, because it suggests the absence of an immediate response. Furthermore we find that in addition to the nature of the background noise, response properties also depend on its amplitude: a certain level of noise optimally promotes the spiking response. Hence noise facilitates the transmission of the input to the output of the neuron. This is stochastic resonance in the general sense of the term as recently suggested [28]. As noted in the introduction, stochastic resonance of the linear response kernel has previously been demonstrated for sinusoidal input currents and Gaussian white background noise [23]. Furthermore, also slow aperiodic transients are facilitated by stochastic resonance in the integrate-and-fire neuron [52]. We extend the known results in two respects. Firstly, we show that the linear response shows aperiodic stochastic resonance also for fast transients. Secondly, we demonstrate that the instantaneous non-linear response exhibits a qualitatively similar, but even more pronounced dependence on noise intensity. For realistically small synaptic amplitudes, the instantaneous non-linear response is typically small compared to the linear contribution. However, this changes at the network level in the presence of feed-forward inhibition: a synchronized pair of an excitatory and an inhibitory pulse evokes spiking responses in two distinct neural populations, whose linear contributions mutually cancel and only the non-linear immediate contribution remains. Hence the immediate response dominates even for small synaptic amplitudes. The presented approximate analytical results are illustrated and confirmed by direct simulation.

The instantaneous non-linear response is potentially a relevant mechanism for processing of transient signals by neurons. In auditory cortex, the irregular firing of neurons has been shown to be driven by simultaneous coactivation of several of their synaptic afferents [56]. The effective postsynaptic potential hence has the amplitude of multiple single synapses, which easily drives the spiking response into the supra-linear regime. The convex increase of firing probability is of advantage to obtain output spikes closely locked to the input. Furthermore, the non-linearity enables the neuron to perform non-trivial computations on the inputs [57]. In particular the memory capacity of networks in a categorization task can be increased by non-linear elements [58]. The circuit presented in section “Dominance of the non-linear component at the network level” establishes a quadratic input-output relationship for fast transient signals that may be useful for non-linear processing, analogous to the non-linear f-I curve (spike frequency as a function of input current) in the case of quasi-stationary rate-coded signals.

Our finding of an immediate non-linear response has an implication on the intensely debated question how common input affects the correlation of the spiking activity of pairs of neurons [30], [31], [59]. The immediate response adds to the correlation at zero time lag, because it increases the probability of both neurons to simultaneously emit a spike. Due to the non-linearity of the mechanism, the immediate firing probability easily becomes the dominant contribution. Our theory yields a means to quantitatively assess this contribution to firing synchrony.

Synapses with spike timing dependent plasticity (STDP) [60] are sensitive to the input-spike triggered firing rate of the neuron. The fast response is relevant, because closely time-locked pre- and postsynaptic activity most effectively changes the synaptic weight. This is illustrated in Figure 1B. The direction of weight change depends on whether the fast response falls on the potentiating or the depotentiating part of the STDP curve, determined by the difference between dendritic and axonal synaptic delay [33]. Assuming that the causal fast response strengthens the synapse (Hebbian learning [61]), the supra-linearity combined with multiplicative spike-timing-dependent learning rules may add new fixed points for the synaptic weight and thus influence pattern formation in recurrent networks [62]. Previous work restricted the analysis of the interplay of neural dynamics and synaptic plasticity in feed-forward [32]–[34] as well as in recurrent networks [35]–[38] to the linear response of the neuron. Our framework extends the scope of analytical investigations of synaptic dynamics to the inherently non-linear response properties of neurons. The pronounced stochastic resonance of the individual neuron implies an optimal level of synaptic background noise that supports cooperativity among afferent synapses and hence also the sensitivity to correlations among them [34]. Measuring synaptic plasticity in the presence of network activity might elucidate how stochastic resonance influences cooperative synaptic learning.

Postsynaptic potentials exhibit a finite rise time, whereas the membrane potential of the integrate-and-fire neuron model jumps at each incoming synaptic event. Although this is a simplification, the model reproduces experimental spike trains surprisingly well [6]. For non-zero rise times, the instantaneous firing rate response reported here is spread out in time over the rising flank of the postsynaptic potential and is proportional to the derivative of the membrane potential [43], [54]. The asymmetry for excitatory and inhibitory synaptic events and the supralinear increase of the response probability with excitatory postsynaptic amplitude, however, are generic features that carry over to finite rise time if the neuron operates in the fluctuation driven regime. Comparing the non-linear and the linear response probability experimentally can serve as an indicator to decide on the importance of each contribution in real neurons. The integral linear response can be obtained from similar arguments as in section “Response to fast transients” as 
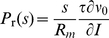
 with the slope of the f-I curve 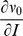 and the membrane resistance 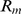.

Previous work has shown that the spike generation mechanism influences the transient properties of neurons [63], [64]. Specifically, a soft threshold, as realized in the exponential integrate-and-fire neuron model [63] is more realistic than the hard threshold of the leaky integrate-and-fire model considered here. Future work needs to investigate how this affects the fast response. We expect qualitatively similar findings, because a positive synaptic impulse shifts membrane potential density into the basin of attraction for spike generation. This will then result in an increased spiking density in a finite time window following the synaptic event.

The hybrid approach combining a diffusion approximation with an explicit treatment of finite jumps near the boundary allowed us to uncover hitherto unknown properties of the integrate-and-fire model by analytical means. The diffusion approximation, however, still limits our approach: for synaptic amplitudes 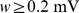 moments of order higher than two, which are neglected by the Fokker-Planck equation, become relevant. A combination of our boundary condition with an assessment of higher moments [19], [65] seems promising. Also, the oscillatory modulations of the probability density on a scale 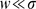 in the regions below threshold and around the reset potential are outside the scope of our theory. The response properties considered in this work are entirely based on the assumption, that the dynamics has reached the steady state prior to arrival of the perturbing input. A valuable future extension of our work is to consider finite amplitude synaptic background noise and additional sinusoidal current injection. This would allow to quantify in a frequency resolved manner how the transfer properties of the model are influenced by finite-grained noise. Technically, the linear perturbation theory for the diffusion limit [22] would have to be combined with our boundary condition. Complications might arise from the fact that the boundary condition is now time-dependent if the mean drive reaches suprathreshold values in certain epochs. Our treatment of stochastic differential equations with finite jumps and absorbing boundaries is general, as long as the jumps are sufficiently small. We expect it to be applicable to other fluctuation driven dynamical systems in quantitative biology and physics. Potential areas include the diffusion of particles in domains with absorbing walls, chemical reactions with activation thresholds, circuit theory and solid state physics.
